# The Medical Association of Georgia

**Published:** 1867-04

**Authors:** 


### THE MEDICAL ASSOCIATION OF GEORGIA.

In the last number of this Journal, we stated that the Medical Association of Georgia would convene in Griffin on the "12th of April." We call attention here, to make the correction. The meeting will take place on the second Wednesday, the 10th of April; and not the "12th of April," as heretofore stated.

In this connection, we would again call the attention of the profession to the importance of a full attendance. Let all attend who can possibly do so.

We learn that the profession in Griffin and vicinity are making preparations to give those in attendance a warm reception, and make their stay pleasant.

From Dr. J. T. Banks, the Chairman of the Committee to select the annual orator, we learn that Dr. V. H. Taliaferro, of Columbus, Ga., will deliver the Annual Address.

